# Validation of Coronary Angiography-Derived Vessel Fractional Flow Reserve in Heart Transplant Patients with Suspected Graft Vasculopathy

**DOI:** 10.3390/diagnostics11101750

**Published:** 2021-09-24

**Authors:** Niya Mileva, Sakura Nagumo, Emanuele Gallinoro, Jeroen Sonck, Sofie Verstreken, Riet Dierkcx, Ward Heggermont, Jozef Bartunek, Marc Goethals, Alex Heyse, Emanuele Barbato, Bernard De Bruyne, Carlos Collet, Marc Vanderheyden

**Affiliations:** 1 Cardiovascular Center Aalst, OLV Clinic, 9300 Aalst, Belgium; nmileva91@gmail.com (N.M.); d04sm059@med.showa-u.ac.jp (S.N.); e.gallinoro@gmail.com (E.G.); jeroensonck@icloud.com (J.S.); sofie.verstreken@olvz-aalst.be (S.V.); riet.dierkcx@olvz-aalst.be (R.D.); ward.heggermont@olvz-aalst.be (W.H.); jozef.bartunek@olvz-aalst.be (J.B.); marc.goethals@olvz-aalst.be (M.G.); alex.heyse@olvz-aalst.be (A.H.); emanuele.barbato@olvz-aalst.be (E.B.); bernard.de.bruyne@olvz-aalst.be (B.D.B.); carlos.collet@gmail.com (C.C.); 2 Cardiology Clinic, Alexandrovska University Hospital, 1430 Sofia, Bulgaria; 3 Department of Cardiology, Showa University Fujigaoka Hospital, Tokyo 8501, Japan; 4 Department of Translational Medical Sciences, University of Campania “Luigi Vanvitelli”, 81100 Naples, Italy; 5 Department of Advanced Biomedical Sciences, University Federico II, 80131 Naples, Italy; 6 Department of Cardiology, Lausanne University Hospital, 1100 Lausanne, Switzerland

**Keywords:** cardiac transplant related arteriopathy, fractional flow reserve, functional assessment

## Abstract

Cardiac transplant-related vasculopathy remains a leading cause of morbidity and mortality in heart transplant (HTx) recipients. Recently, coronary angiography-derived vessel fractional flow reserve (vFFR) has emerged as a new diagnostic computational tool to functionally evaluate the severity of coronary artery disease. Although vFFR estimates have been shown to perform well against invasive FFR in atherosclerotic coronary artery disease, data on the use of vFFR in heart transplant recipients suffering from cardiac transplant-related arteriopathy are lacking. The aim of the presented study was to validate coronary angiography-derived vessel fractional flow reserve to calculate fractional flow reserve in HTx patients with and without cardiac transplant-related vasculopathy. A prospective, single center study of HTx patients referred for annual check-up, undergoing surveillance coronarography was conducted. Invasive FFR was measured using a motorized device at the speed of 1.0 mm/s in all three major coronary arteries. Angiography-derived pullback FFR was derived from the angiogram and compared with invasive FFR pullback curve. Overall, 18,059 FFR values were extracted from the FFR pullback curves from 23 HTx patients. The mean age was 59.3 ± 9.7 years, the mean time after transplantation was 5.24 years [IQR 1.20, 11.25]. A total of 39 vessels from 23 patients (24 LAD, 11 LCX, 4 RCA) were analyzed. Mean distal vFFR was 0.87 ± 0.14 whereas invasive distal FFR was 0.88 ± 0.17. An excellent correlation was found between invasive distal FFR and vFFR (r = 0.92; *p* < 0.001). The correlation of the pullback tracing was high, with a correlation coefficient between vFFR and invasive FFR pullback values of 0.72 (95% CI 0.71 to 0.73, *p* < 0.001). The mean difference between vFFR and invasive FFR pullback values was −0.01 with 0.06 of SD (limits of agreements −0.12 to 0.13). In HTx patients, coronary angiography-derived FFR correlates excellently with invasively measured wire-derived FFR. Therefore, angiography derived FFR could be used as a novel diagnostic tool to quantify the functional severity of graft vasculopathy.

## 1. Introduction

Cardiac transplant-related vasculopathy (CAV) is caused by alloimmune and non-immune mechanisms that can cause intimal hyperplasia and usually manifests as diffuse concentric luminal narrowing. According to data of the international society for heart and lung transplantation (ISHLT) registry, its incidence as detected by invasive coronary angiography is 7.8%, 29.3% and 47.4% at 1, 5 and 10 years, respectively [1]. Therefore, graft failure due to CAV remains a leading cause of late mortality in this population [2]. In recent years, fractional flow reserve derived from coronary angiography (vFFR) has emerged as a new diagnostic computational tool to functionally evaluate the severity of coronary artery disease, and vFFR estimates have been shown to perform well against invasive FFR in atherosclerotic coronary artery disease [3,4]. Furthermore, we recently showed that the functional assessment of the coronary circulation using the angiography derived FFR (vFFR) is feasible in heart transplant recipients [5]. However, to the best of our knowledge, direct comparison of vFFR with invasive FFR measurements in HTx patients has not been performed yet. Therefore, the aim of the present study was to evaluate the angiography-derived vessel FFR in heart-transplant patients with and without cardiac transplant-related arteriopathy using invasive FFR as reference

## 2. Materials and Methods

### 2.1. Study Design

This is a prospective, single center, investigator-initiated observational study. The study population consisted of heart transplant recipients referred for an annual suveillance coronary angiography. The presence and extent of cardiac allograft vasculopathy was graded according to the standard ISHLT criteria, as previously described [6]. Briefly, CAV was classified as absent (CAV 0), mild (CAV 1), moderate (CAV 2), or severe (CAV 3) according to the ISHLT classification. The study protocol was approved by the investigational review board of the ethics committee. All patients signed informed consent before the study procedures. 

### 2.2. Invasive Procedure

Following the administration of nitroglycerine intracoronaryly, coronary angiograms were acquired in two projections separated by at least 30 degrees of difference in angulation. FFR measurements were performed following the recommendations of the Standardization of Fractional Flow Reserve Measurements document [7]. Briefly, the pressure wire sensor was positioned in the distal coronary segments of >2 mm of diameter by visual estimation. Pressure wire position was recorded using contrast injection to identify the pullback start position. A continuous intravenous adenosine infusion was given at a dose of 140 mcg/kg/min via a peripheral or central vein to obtain steady-state hyperemia for at least 2 min. A pullback device (Volcano R 100, San Diego, CA, USA), adapted to grip the coronary pressure wire (PressureWire X, St. Jude Medical, MN, USA), was set at a speed of 1 mm/s to pull back the pressure wire sensor up to the tip of the guiding catheter during continuous pressure recording. The maximal pullback length was 130 mm per vessel. If FFR drift (>0.03) was observed, the FFR pullback was repeated. FFR was defined as the lowest ratio between distal and proximal coronary pressures during hyperemia. 

### 2.3. Angiography-Derived FFR Computation

Angiography-derived fractional flow reserve analysis was performed using a validated software (vFFR, CAAS 8.2 Workstation, Pie Medical Imaging, Maastricht, The Netherlands) [4]. The vFFR workflow builds a 3D reconstruction of the coronary artery and calculates pressure drop and a FFR value (vFFR) by applying physical laws, which include viscous resistance and separation loss effects to the 3D coronary reconstruction [3]. The procedure for vFFR calculation can be summarized as follows. Following automatic calibration, based on the geometrical data stored in the Digital Imaging and Communications in Medicine (DICOM) headers, two angiographic projections with a difference in angulation or rotation of ≥30 degrees are selected. The end-diastolic frames of the selected angiograms, filled with adequate contrast, are synchronized with each other either manually or automatically by ECG gating. In both images, a path of centerline points is placed through the vessel of interest which enables the automatic contour detection of the luminal borders of the vessel. Simultaneously, a common image point is selected at similar positions in both projections, using anatomical landmarks. If needed, contour corrections are allowed. Finally, the vessel model is automatically reconstructed in 3D space assuming an elliptical cross-sectional shape, and the pressure drop along with vFFR values are calculated by a proprietary algorithm. (Suppl. Figure S1) VFFR values ≤0.80 are considered as significant disease. To create the virtual pullback curve, vFFR values at every 1 mm were extracted and plotted along the length of the vessel. Values between non-invasive and invasive evaluations were matched using anatomical landmarks, namely, the distal position of the pressure wire and the coronary ostium.

### 2.4. Study Objectives

The primary objective of this study is to validate coronary angiogram-derived vessel fractional flow reserve to calculate fractional flow reserve in HTx patients with and without cardiac transplant-related vasculopathy.

### 2.5. Statistical Analysis 

Continuous variables with normal distribution are presented as mean ± SD; whereas non-normally distributed variables are presented as median (interquartile range). Categorical variables are presented as counts and percentages. The primary objective is the agreement between the vFFR and the invasively measured FFR assessed using the Bland–Altman method [8]. The mean difference is considered a metric of accuracy, and the standard deviation of the mean difference as a metric of precision. All analyses are performed using R statistical software version 3.5.3 (R Foundation for Statistical Computing, Vienna, Austria).

## 3. Results

**Figure 1:**
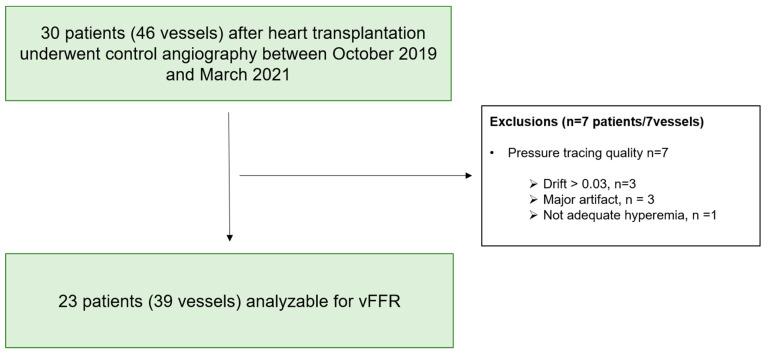
Patient selection flow chart. vFFR—angiography derived fractional flow reserve.

Figure 1 depicts the flow diagram illustrating the selection of the study population. From October 2019 to March 2021, 29 vessels of 39 heart transplant patients who underwent annual surveillance coronary angiography were selected for vFFR and FFR analysis. In 3 patients, 2 vessels were analyzed; whereas in the rest, FFR and vFFR were assessed in one coronary territory. The clinical characteristics of the study population are summarized in Table 1. 87% (n = 20) of the final cohort were male, mean age was 59.2 ± 9.7 years, and the median time between transplantation and coronary angiography was 5.24 years [IQR 1.20–11.25]. Anatomical vessel characteristics are summarized in Table 2. Mean minimum lumen diameter (MLD) was 1.94 ± 0.69 mm with mean reference vessel diameter of 2.90 ± 0.81 resulting in a mean %DS of 32.5 ± 16% diameter stenosis. According to the CAV ISHLT classification, 10 patients had CAV 0 and 13 patients CAV ≥ 1 grade (CAV 1 grade: 8 pts, CAV 2 grade: 4 pts, CAV 3 grade: 1 patient). 

### Functional Evaluation

**Figure 2:**
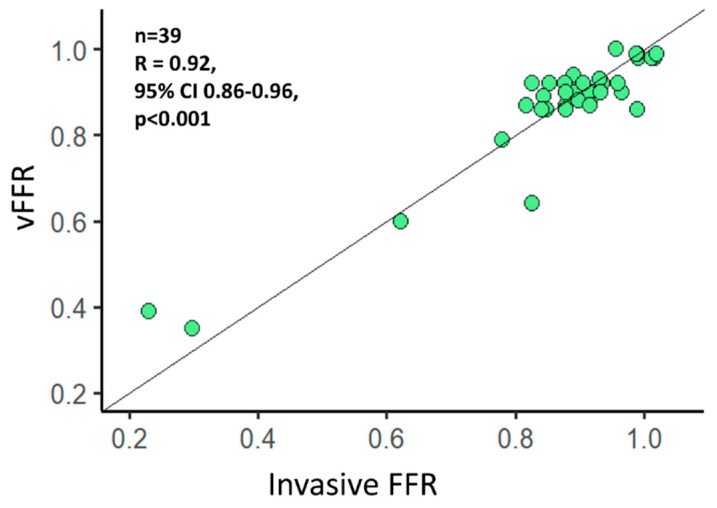
Correlation between angiography-derived vessel fractional flow reserve (vFFR) and invasive FFR. Pearson correlation index of 0.92, 95% CI 0.86–0.96, *p* < 0.001.

**Figure 3:**
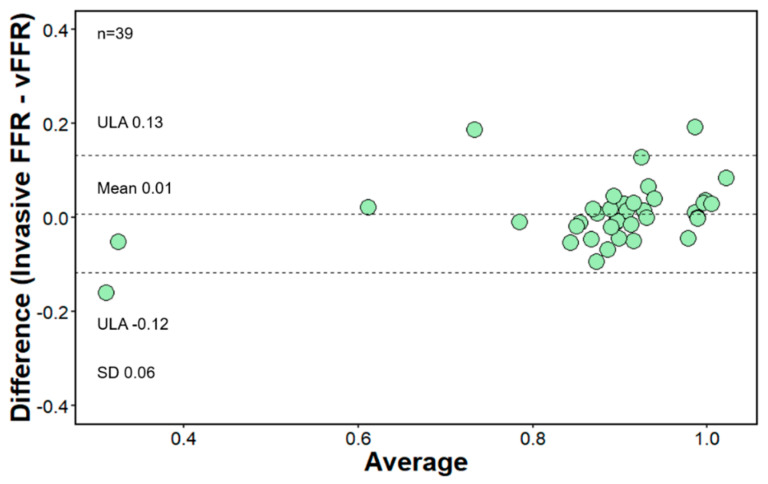
Bland−Altman plot-agreement between invasive FFR and angiography-derived FFR (vFFR). Mean difference 0.01 FFR units, SD ± 0.06, limits of agreement −0.12 to 0.13.

**Figure 4:**
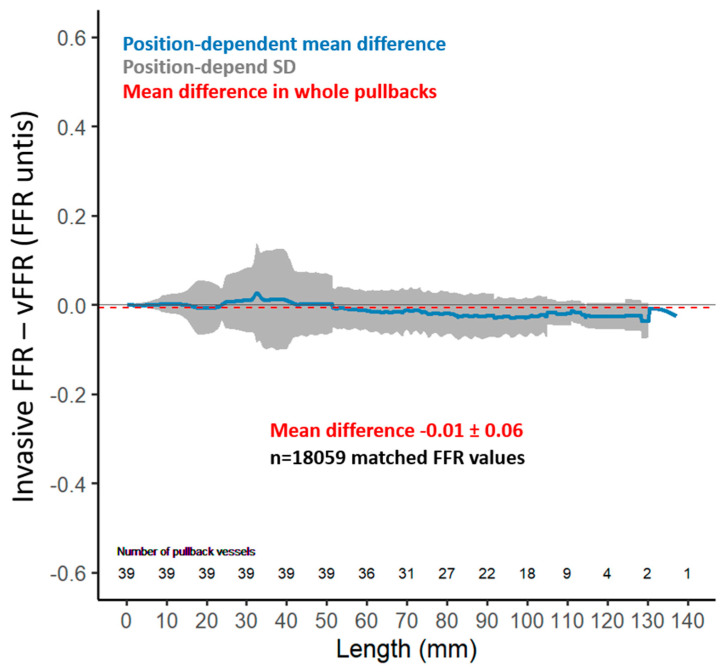
Bland−Altman plot-agreement between invasive FFR and angiography-derived FFR (vFFR) pullbacks. Mean difference −0.01 FFR units, SD ± 0.06.

A total of 39 vessels (24 LAD, 11 LCX, 4 RCA) were analyzed, and overall, 18059 FFR values were extracted from the FFR pullback curves. The functional vessel characteristics are summarized in Table 2. Mean distal vFFR was 0.87 ± 0.14 whereas invasive distal FFR was 0.88 ± 0.17 with 12.8% having a vFFR ≤ 0.80 and 10.3% having a FFR ≤ 0.80. An excellent correlation was found between invasive distal FFR and vFFR (r = 0.92; *p* < 0.001) (Figure 2). Furthermore, agreement between invasive FFR and vFFR was excellent, with a mean difference 0.01 FFR units, SD ± 0.06, and limits of agreement −0.12 to 0.13 (Figure 3). The correlation of the pullback tracing was high, with a correlation coefficient between vFFR and invasive FFR pullback values of 0.72 (95% CI 0.71 to 0.73, *p* < 0.001). The mean difference between vFFR and invasive FFR pullback values was −0.01 with 0.06 of SD (limits of agreements were −0.12 to 0.13 [Figure 4]). Also, in those with angiographic evidence of CAV defined by an ISHLT CAV ≥ 1 score, a good correlation was noted between invasively measured distal FFR and vFFR (r = 0.691; *p* < 0.001; [Figure S2]) with a mean difference between vFFR and invasive FFR of −0.01 and SD of 0.04 (limits of agreements were −0.07 to 0.05).

## 4. Discussion

The main findings of the present study can be summarized as follows: (i) functional evaluation of coronary arteries in heart transplant patients based on angiography-derived vessel fractional flow reserve measurement is feasible; (ii) a good correlation and an excellent agreement is observed between the angiography-derived FFR and invasively measured FFR.

As a result of more advanced immunosuppressive therapies and better surgical techniques, short-term survival after cardiac transplantation has improved substantially. However, long-term survival is limited by significant mortality rates beyond the first year after transplant, which have unfortunately remained constant for the past 2 decades [9]. CAV affects over 50% of the recipients by 10 years after transplant and represents one of the important causes responsible for this high mortality. Opposite to the focal epicardial lesions in atherosclerosis, CAV is characterised by a progressive, diffuse concentric intimal hyperplasia involving both the epicardial and intramyocardial arteries. Multifactorial immunologic and nonimmunologic factors of both the donor and the recipient play a major role in the pathogenesis of the disease [10]. In contrast to atherosclerosis, CAV is difficult to diagnose in its early stages as it is typically silent in the denervated heart, and ischemia or graft dysfunction are usually not evident until the disease is advanced and manifests as heart failure, arrhythmias, or sudden death. [6] Currently annual surveillance coronary angiography is recommended as the screening tool for CAV by the International Society of Heart and Lung Transplantation (ISHLT) [6]. However, as a consequence of the typical concentric vascular remodelling process and its low-resolution, coronary angiography often underestimates the severity and extent of CAV. By imaging the vessel wall structure, coronary IVUS is able to detect and quantify the intimal hyperplasia and partly overcomes these limitations, making it so far the most sensitive test for the detection and risk stratification of CAV [11]. However, IVUS is not routinely used in clinical practice due to its invasiveness, lack of widespread expertise, and higher costs. In addition, it only allows the assessment of the proximal epicardial arteries. 

In this regard, the functional evaluation of the coronary arteries by fractional flow reserve (FFR), has emerged as a new invasive tool to predict adverse clinical outcomes in heart transplant recipients [12,13]. In CAV, FFR correlates with IVUS assessed plaque volume and a pathological FFR has been observed in a significant proportion of asymptomatic cardiac transplant patients with normal angiograms [12]. In line with these observations, we recently reported a similar functional coronary impairment undetectable by standard anatomical analysis in up to 32% of heart-transplant patients [5].

While the gold-standard invasive FFR requires the induction of hyperaemia and thus is related to prolonged procedural time and patient discomfort, the angiography-based FFR has the advantage that it can be computed from a 3-dimensional reconstruction of the coronary artery obtained from conventional invasive coronary angiography using computational fluid dynamics calculations or by a mathematical approach. In patients with atherosclerotic coronary artery disease, an excellent correlation has been reported between angiography-derived FFR and invasive FFR [8,9]. 

The mean difference between vFFR and invasive FFR in our study was 0.01 FFR units, SD ± 0.06, with limits of agreement from −0.12 to 0.13. Therefore, we hypothesize that angiography-derived vessel fractional flow reserve may be a helpful tool in the evaluation of cardiac allograft vasculopathy and could play an important role in risk stratification after heart transplantation. The novelty of our study is similar to what has been observed in atherosclerotic heart disease also in CAV; the angiography-derived FFR can be used as a less invasive counterpart of FFR as demonstrated by the excellent agreement between both parameters. Important to note is the fact that even though the correlation between the invasive FFR and vFFR is less good in patients with higher grade of CAV, with a mean difference between vFFR and invasive FFR of -0.01 (SD of 0.04), the accuracy of the method in patients with CAV ≥ 1 remains high. This opens new perspectives for a widespread and easily acquired method for precise functional assessment of graft vasculopathy and may help in achieving an earlier diagnosis and a better management of the disease, resulting in higher long-term survival rates.

### Limitations

The present study has several limitations. First, this study is limited by its observational single center approach and by the small number of patients included in the final analysis. Secondly, we only evaluated CAV by coronary angiography, which is less sensitive to detect early stages of CAV than intravascular ultrasound or optical coherence tomography. However, this grading system is the standard method for grading the extent of CAV (1). Finally, no follow-up data for MACE was evaluated as it was not the study objective. Therefore, further studies for assessment of clinical outcomes of CAV detected using angiography-derived FFR are warranted for this purpose. Furthermore, previous analysis has shown that a cut-off value of 0.90 for invasive FFR is a predictor of mortality in heart transplanted patients [14]. Hence, future studies are needed to determine the specific threshold of vFFR that is associated with adverse clinical outcomes in heart transplanted patients.

## 5. Conclusions

In HTx patients, coronary angiography-derived FFR correlates excellent with invasively measured wire-derived FFR and could therefore be considered as an alternative for grading the extent of CAV. Similar to the invasive FFR, angiography derived FFR is a novel diagnostic tool providing information about the functional severity of graft vasculopathy and might be helpful in finetuning the management of these patients. Further prospective studies that explore the role of angiography derived FFR in grading CAV severity are warranted.

## Figures and Tables

**Table 1:** Patient demographic and clinical characteristics.

Variables	Overall
Total number of patients, *n*	23
Age receptor at angiography, years (mean (SD))	59.30 (9.69)
Sex, Male, *n* (%)	20 (87.0)
Dyslipidemia, *n* (%)	22 (95.7)
Hypertension, *n* (%)	9 (39.1)
Diabetes mellitus, *n* (%)	10 (43.5)
Current smoker, *n* (%)	1 (4.3)
History of ischemic cardiomyopathy, *n* (%)	11 (47.8)
Other cardiomyopathies, *n* (%)	12 (52.2)
Time between heart transplant and coronary angiography, years (median [IQR])	5.24 [1.20, 11.25]
CAV ≥ 1 grade, *n* (%)	13 (56.5)

SD—standard deviation; IQR—interquartile range; CAV—Cardiac allograft vasculopathy.

**Table 2:** Patient procedural characteristics.

Variables	Overall
Total number of vessels, *n*	39
Type of vessel, *n* (%)	
LAD, n (%)	24 (61.5)
LCX, n (%)	11 (28.2)
RCA, n (%)	4 (10.3)
Invasive FFR, (mean (SD))	0.88 (0.17)
Invasive FFR ≤ 0.80, *n* (%)	4 (10.3)
Lesion length, mm, (mean (SD))	23.0 (14.3)
Minimal lumen diameter, mm, (mean (SD))	1.94 (0.69)
Diameter stenosis, %, (mean (SD))	32.5 (16.0)
Reference vessel diameter, mm, (mean (SD))	2.90 (0.81)
vFFR, (mean (SD))	0.87 (0.14)
vFFR value ≤ 0.80, *n* (%)	5 (12.8)

LAD—left anterior descending artery; LCX—left circumflex artery; RCA—right coronary artery; FFR—fractional flow reserve; vFFR—angiography-derived fractional flow reserve.

## Data Availability

The data presented in this study are available on request from the corresponding author.

## Supplementary Materials

The following are available online at https://www.mdpi.com/article/10.3390/diagnostics11101750/s1. Figure S1: Coronary angiography images of LAD with illustration of invasive motorized FFR pullback with distal value of FFR 0.61 and angiography-derived FFR pullback with distal FFR value of 0.60. Figure S2: Correlation between angiography-derived vessel fractional flow reserve (vFFR) and invasive FFR. Pearson correlation index of 0.92, 95\% CI 0.86-0.96, *p* < 0,001 with color coding based on the cardiac allograft vasculopathy (CAV) classes.
