# Supplementary material for: Validation of Coronary Angiography-Derived Vessel Fractional Flow Reserve in Heart Transplant Patients with Suspected Graft Vasculopathy
Source: Diagnostics (Basel). 2021 Sep 24;11(10):1750. doi: 10.3390/diagnostics11101750 (PMC8534544; doi:10.3390/diagnostics11101750)
Supplement: Supplementary file 1 [file diagnostics-11-01750-s001.zip › diagnostics-1312402-Supplementary.pdf]

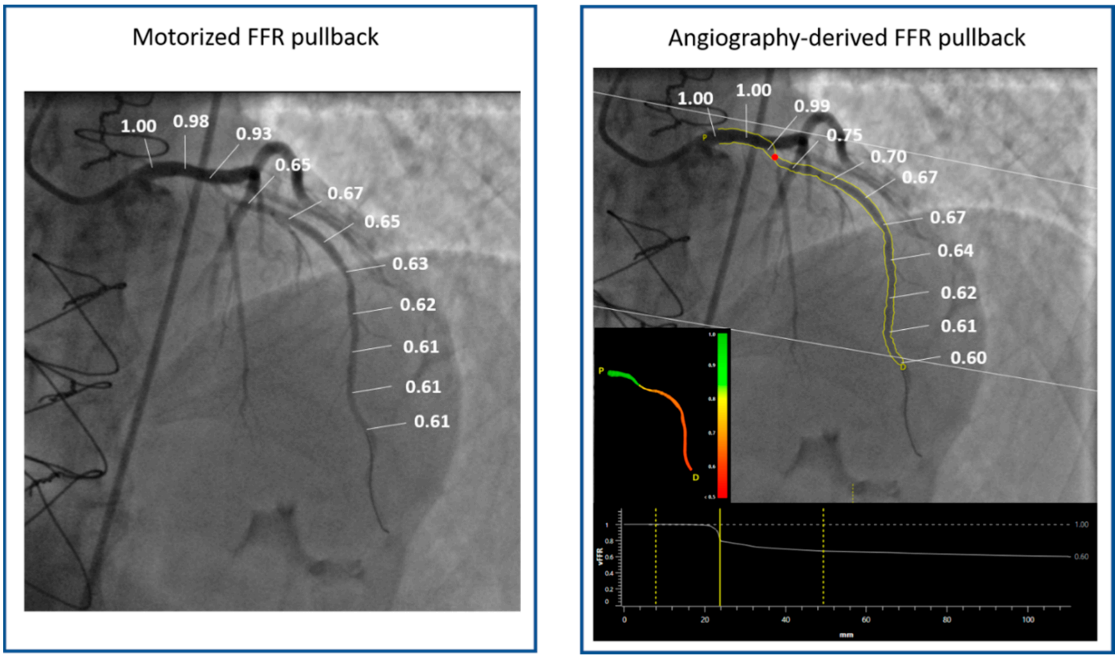

LAD – left anterior descending artery. FFR – fractional flow reserve.

Figure S1. Coronary angiography images of LAD with illustration of invasive motorized FFR pullback with distal value of FFR 0.61 and angiography-derived FFR pullback with distal FFR value of 0.60.

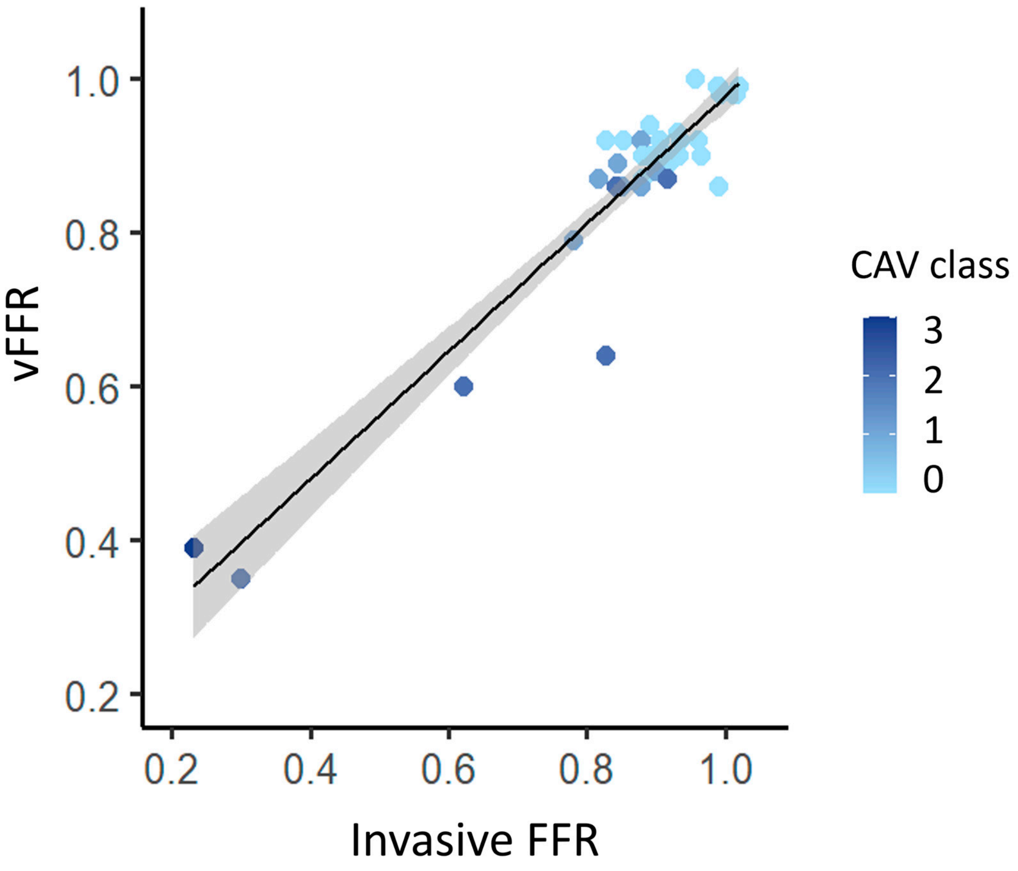

Figure S2. Correlation between angiography-derived vessel fractional flow reserve (vFFR) and invasive FFR. Pearson correlation index of 0.92, 95% CI 0.86-0.96, $p < 0{,}001$ with color coding based on the cardiac allograft vasculopathy (CAV) classes.
